# H-Bonds Enhanced Natural Polyphenols Bined Polysaccharide/Gelatin Composites with Controlled Photothermal Stimulation Phase Transition for Wound Care

**DOI:** 10.34133/bmr.0082

**Published:** 2024-09-13

**Authors:** Chonghao Chen, Junbo Zhang, Guofeng Zhong, Pengkun Lei, Xuhua Qin, Chen Zhang, Rui Zeng, Yan Qu

**Affiliations:** ^1^ State Key Laboratory of Southwestern Chinese Medicine Resources, School of Pharmacy, Chengdu University of Traditional Chinese Medicine, Chengdu, Sichuan 611137, China.; ^2^ State Key Laboratory of Quality Research in Chinese Medicine, Institute of Chinese Medical Sciences, University of Macau, Macau, China.; ^3^ College of Pharmacy, Southwest Minzu University, Chengdu 610041, China.; ^4^ Key Laboratory of Research and Application of Ethnic Medicine Processing and Preparation on the Qinghai Tibet Plateau, Chengdu 610041, China.

## Abstract

Severe open wounds should be closed immediately and regularly undergo re-examination and debridement. Therefore, dressings should effectively cover the wound, creating a moist environment for healing while meeting mechanical requirements for daily movement and adaptability. Herein, a low-cost and easy-to-prepare plant polysaccharide hydrogel was reported. The *Mesona chinensis* Benth polysaccharide strengthened the hydrogel network by hydrogen bonding and changed the phase transition temperature, but retained the thermal response characteristics of the hydrogel. By adjusting the polysaccharide concentration, MepGel(1) can be prepared to remain stable as a semisolid at body temperature and transform into a shear-thinning semifluid state when appropriately heated. The composite hydrogel could be easily shaped, effectively closing wounds of different shapes, while maintaining excellent mechanical properties. Importantly, this composite hydrogel had a near-infrared photothermal effect resulting in excellent antibacterial effect and collided with its own thermal response producing functions conducive to wound care, like accelerating the self-healing of the dressing, achieving re-adhesion, and further covering the wound. Furthermore, the hydrogel had excellent biocompatibility, enhancing immunity and promoting healing of bacterial-infected wounds. The low cost and rich functionality demonstrated by MepGel had the potential to face the enormous challenges and economic burden of clinical wound healing.

## Introduction

Wounds are a common disease but face severe challenges and bear a serious economic burden [1]. One of the challenges is wound infection, usually caused by patients’ untimely and inappropriate treatment, and is becoming increasingly severe due to obstacles in the development and application of antibiotics [2]. In general, severe open wounds should be debrided and closed immediately to prevent bacterial invasion [3]. Moreover, in the coming weeks, the wound may require repeated observation, debridement, and closure to provide a suitable healing environment [4,5]. Therefore, the ideal wound dressing should be low cost and easy to prepare. It can effectively close the wound while avoiding the safety and mechanical deficiencies like injectable wound dressings often present. Furthermore, in the future process of wound healing, wound dressings are required to be easily manipulated for closure and unloading, providing convenience for daily wound care.

In recent decades, non-antibiotic antibiotics have always been the focus of basic medical research [6]. Near-infrared (NIR) photothermal therapy has attracted much attention in antimicrobial applications due to its advantages such as remote control, deep penetration into tissues, immediacy, broad-spectrum antibacterial effect, and low bacterial resistance [7,8]. A large number of NIR photothermal materials such as (non-)/metallic compounds, noble metal nanoparticles, and organic dye have been reported [9]. However, these materials have problems such as high inherent cost, poor biocompatibility, cumbersome synthesis, and involving environmentally unfriendly organic reagents, which hinder the transformation from research to application [10,11]. Therefore, researchers have begun to explore photothermal materials from readily available biological sources. Zhang and colleagues [12] reported on the photothermal effects of natural nanoparticles rich in amino acids and polysaccharides extracted from cuttlefish juice, and Yu and colleagues [13] further modified and loaded this nanoparticle into microneedles for the treatment of skin tumors and wound healing. Nevertheless, the benefits of a simple addition of photothermal materials for wound dressings are limited to the antibacterial function. Ideally, photothermal materials should be used as part of the material to optimize the physical properties of the material and, in combination with photothermal effects to expand the material’s functionality, to meet the challenges of daily care and unexpected situations.

*Mesona chinensis* Benth (MCB), an annual herb of Labiatae, is rich in polysaccharides, polyphenols, and flavonoids, with a variety of biological activities such as anti-oxidation and anti-inflammatory effects [14]. Currently, as an important medicinal and edible plant resource, it is widely used in the preparation of herbal tea and food hydrogels [15]. Previous investigations have revealed that, as the main component of MCB, the polysaccharide of MCB (MCBP) is a polysaccharide that naturally binds a mixture of brown bioactive compounds such as polyphenols and has special gel characteristics [16]. Through noncovalent bond interaction, MCBP can form hydrogels with a variety of polysaccharides or proteins, improving the mechanical properties of hydrogels, and possibly endowing them with thermal reversible properties [17,18]. Meanwhile, MCBP can regulate the activity of macrophages and T lymphocytes, identify and eliminate foreign pathogenic microorganisms, and effectively eliminate superoxide anion and other free radicals to maintain the stability of the internal environment [19,20]. Importantly, MCBP has a relatively broad absorption spectrum, which has attracted growing interest in absorbing ultraviolet (UV) radiation and showed the potential for NIR photothermal conversion [21].

**Fig. 1.**
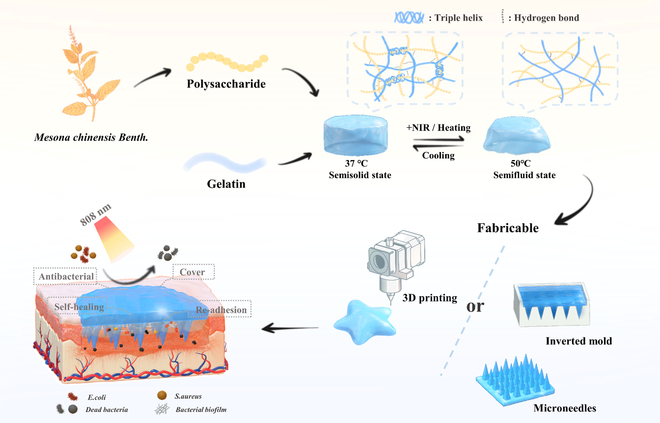
Schematic explanation of the preparation of MepGel and their thermal reversibility, shape plasticity, and application as wound dressing materials.

Therefore, as shown in Fig. 1, a NIR photothermal-responsive natural hydrogel (MepGel) was developed by simply mixing MCBP and gelatin to meet the need for a wound sterile environment and daily wound care. The cross-linking between MCBP and gelatin via hydrogen bond led to the increase of phase transition temperature of this polysaccharide/gelatin composite. The morphological and physical properties of hydrogels with different amounts of MCBP were evaluated, and the ideal MCBP concentration for wound care dressing was screened out. The MepGel hydrogel prepared at this concentration presented a stable semisolid (SS) hydrogel state at body temperature and a semifluid (SF) weak hydrogel state with shear-thinning characteristics at 50 °C or under NIR irradiation. Benefiting from the 2 states, this natural hydrogel can prepare various fine 3-dimensional (3D) structures with excellent mechanical properties through inverted molding or extrusion 3D printing to match wounds of various shapes and depths. Combined with its weak bond cross-linking, the photothermal effect will quickly break the original hydrogel network and then reassemble after cooling. Therefore, we evaluated the NIR stimulus responsiveness of this hydrogel, such as acceleration of self-healing, promotion of wound coverage, and restart adhesion, which provided convenient daily care for the wound. In addition, biological experiments evaluated the antibacterial effect, biocompatibility, macrophage activation, and impact on wound healing of the MepGel hydrogel. The MepGel hydrogel has potential application in clinical wound healing and nursing. Meanwhile, due to the abundant resources of MCB and the simple preparation process, MepGel also demonstrated great potential for clinical transformation.

## Materials and Methods

### Materials and animals

Gelatin from porcine skin, type A, was purchased from Sigma-Aldrich, Germany. Na_2_CO_3_ and all other chemicals were of analytical grades and purchased from Shanghai Macklin Biochemical Co. Ltd. Cell Counting Kit-8 (CCK-8), Live-Dead Cell Staining Kit, Dulbecco’s modified Eagle’s medium (DMEM), fetal bovine serum (FBS), and phosphate-buffered saline (PBS; pH 7.4) were purchased from Thermofisher Biochemical Products Co. Ltd.

Dried *M. chinensis* herb was purchased from Wuping, Longyan, Fujian. MCBP was prepared based on previous reports [22,23]. Briefly, MCB was ground into powder, soaked in ethanol for 24 h, filtered, and dried. The obtained powder was degreased twice with ethanol and petroleum ether for 2 h, and dried. The powder was mixed with 0.5% Na_2_CO_3_ (w/v, milli-Q) solution, stirred, and heated at 90 °C for 2.5 h. The obtained solution was centrifuged and collected the filtrate, adjusted the pH to 7, and concentrated and precipitated with ethanol.The precipitates were collected and washed with ethanol, acetone, and anhydrous ether. After drying, MCBP was obtained by dialysis with milli-Q water for 3 d and freeze-drying.

All animals were purchased from SPF (Beijing) Biotechnology Co. Ltd. All animal experiments were approved by the Experimental Animal Protection Association of Chengdu University of Traditional Chinese Medicine, following the *Guidelines for the Care and Use of Laboratory Animals* published by the National Institutes of Health, USA. The license for the Use of Laboratory Animals is No. SYXK2020-124.

### Preparation of sample

MCBP was dissolved in 0.2% Na_2_CO_3_ (w/v, milli-Q) solution. After MCBP was dissolved, the solution was heated to 60 °C and 10% (w/v) gelatin was added. After the gelatin was fully mixed with MCBP, it was cooled to obtain the MepGel hydrogel. The contents of MCBP in MepGel(1), MepGel(0.5), and MepGel(2) were 1%, 0.5%, and 2% (w/v), respectively. The MepGel(1) hydrogel pre-solution before cooling was used as the 3D printing ink. The temperature of the printing syringe was set at 50 °C, and the temperature of the receiving platform was room temperature. In addition, the pre-solution was centrifuged into a well-designed mold, cooled, and dried at room temperature for 2 d, and the MepGel(1) microneedle patch was obtained after demolding. Gelatin hydrogels and microneedles were prepared by the same method without MCBP. Gelatin/alginate/Ca^2+^ IPN (interpenetrating polymer network) hydrogel was prepared by mixing gelatin and alginate at 60 °C and crosslinking with 100 mM CaCl_2_ solution for 3 h after cooling.

### Characterization

#### Morphology of the MepGel hydrogel (SEM)

The morphology of the different hydrogels was observed by using a scanning electron microscope (SEM; SU9010, Hitachi). The hydrogel samples were freeze dried, and the cross-sections were observed after vacuum sputtering and gilding.

#### The compressive strength of the MepGel hydrogel

The compressive strength was measured by a texture analyzer (rapid TA +, Shanghai Tengbo instrument). Briefly, the hydrogels were prepared in a cylindrical shape with a diameter of 14 mm and a height of 1.2 cm. Then, the texture analyzer was used to test at the speed of 1 mm/min.

#### The adhesion strength of the MepGel hydrogel

The adhesion strength (lap shear test) was measured by a texture analyzer (rapid TA +, Shanghai Tengbo instrument). The lap shear test was used to test the bonding strength of the hydrogels. Briefly, the fresh pigskin was cut into a rectangle (50 × 20 mm^2^) and put into distilled water. The hydrogel is transferred to the subcutaneous tissue of the pigskin where excess surface water was removed by filter paper. Then, another pigskin was placed on it with a bonding area of 10 × 20 mm^2^. After the gel was stabilized, the adhesive strength was measured with the texture analyzer at a tensile rate of 5 mm/min^−1^.

#### The swelling degree of the MepGel hydrogel

The swelling ratio of each hydrogel sample [including MepGel(1), MepGel(0.5), MepGel(2), and Gelatin] was determined by the following methods. Briefly, once formed, the hydrogel was lyophilized and weighed (*W*_*d*_). Subsequently, the hydrogel samples were placed in 1 mM PBS at room temperature. The hydrogel was removed from time to time, filtered the water on the surface of the hydrogel, and weighed (*W*_*t*_). Finally, the weight of the hydrogel no longer changes, and it reaches the equilibrium swelling state. The swelling ratio of the hydrogel at a different time was calculated by the following formula.

$$\text{Swelling ratio}=(W_{t}-W_{d})/W_{d}\times 100\%$$

where *W*_*t*_ is the mass of the swelling hydrogel and *W*_*d*_ is the mass of the dry hydrogel.

#### Fourier transform infrared spectroscopy

Fourier transform infrared (FTIR) spectra were assayed using an FTIR equipped with attenuated total reflectance accessory (ATR-FTIR, Agilent Cary 610). The samples were prepared by the KBr disk method. ATR (attenuated total refraction) mode was applied to the MepGel hydrogel. All spectra were obtained by recording between 400 and 4,000 cm^−1^ at a resolution of 4 cm^−1^ up to 64 scans.

#### Rheological characterization

The rheological properties of the hydrogel were determined using TA Instruments DHR-2 rheometer with a 40-mm test plate. After loading the sample onto the Peltier plate, the side of the plate was coated with low-viscosity mineral oil to prevent water evaporation. (a) The oscillating frequency was measured in the range of 0.1% to 100% angular frequency at 25 °C and 1% strain. (b) The temperature sweep was measured from 15 to 50 °C at 1% strain and 1 Hz. (c) At 1 Hz, oscillation amplitude sweep measurements with shear strain ranging from 0.1% to 1,000% were carried out at 37 and 50 °C. (d) Oscillating time scanning experiments were performed with alternating small oscillatory force (γ = 1% strain) and large oscillatory force (γ = 1,000% strain) at 1 Hz and 50 °C. (e) The flow temperature ramp testing of 3 cooling/heating cycles was measured at 1 1/s shear rate. The rate of temperature change was 5 °C/min.

### Photothermal performance

The cylindrical hydrogel sample (diameter: 8 mm, height: 3 mm) was placed in a transparent container and continuously irradiated with an 808-nm laser (2 W/cm^2^) for 1 min. An infrared thermal imaging camera was used to photograph temperature changes during the process. In addition, the samples underwent 4 NIR irradiation heating and natural cooling cycles to verify the repeatable photothermal properties of the samples.

### Cytocompatibility tests

#### CCK-8 studies

The coculture medium of hydrogel and medium was used to evaluate the cytotoxicity of hydrogels. The sterilized samples were added to DMEM (10% FBS–1%penicillin-streptomycin), cultured at 37 °C for 24 h, and configured with different concentrations of coculture solution. L929 cell suspension (2 × 10^4^ cells/ml) was inoculated on a 96-well plate, cultured in a 5% CO_2_ incubator at 37 °C for 12 h, and then changed to a coculture medium for 48 h. Cytotoxicity was then determined by CCK-8 according to the manufacturer’s instructions.

#### Live-dead cell staining analysis

The direct contact method was used to further determine the cytotoxicity of hydrogels to L929 cells. Briefly, the MepGel(1) hydrogel was filled into the bottom of the confocal culture dish and then sterilized after gel. A cell-free medium was added to a confocal culture dish and balanced for 30 min. Then, the culture medium was removed and the L929 cells (1 × 10^5^ cells per well) were added to the confocal culture dish. After incubating with the MepGel(1) hydrogel for 1 to 3 d, the cells were stained with a Live-Dead Cell Staining Kit according to the manufacturer’s instructions. After calcein-AM staining, the cell morphology was observed with a fluorescence microscope (IX73, Olympus).

#### Quantitative real-time PCR assay

Raw 264.7 cells were cultured on the samples at a density of 1 × 10^5^ cells/ml and cultured at 37 °C for 24 h. RNA was extracted, and real-time quantitative polymerase chain reaction (qPCR) was performed using GoTaq qPCR Master Mix reagent and Mx3005P qPCR systems according to the manufacturer’s guidelines.

### In vitro antibacterial tests

The hydrogel sample (diameter: 8 mm, height: 3 mm) was placed at the bottom of the 48-well plate. *Staphylococcus aureus* (or *Escherichia coli*) suspension (200 μl) [10^6^ colony-forming units (CFU/ml] was added to the corresponding wells, and after standing for 5 min, it was irradiated with NIR (2 W/cm^2^). The irradiation time was 1 min, and 3 heating and cooling cycles were conducted. After irradiation treatment, 800 μl of medium was added and then cultured at 37 °C for 12 h. Finally, the number of bacteria was studied by dilution plate counting method and the bacterial viability was calculated. The blank group did not contain any samples, and all the experimental groups without irradiation were set as references.

$$\begin{array}{c} \text{Bacterial viability}={\text{Sample group}}_{\text{Number of bacteria}}/\\ \text{Blank}\;{\text{group}}_{\text{Number of bacteria}}\times 100\% \end{array}$$

### Bacterial erosion tests

 Hydrogel pre-solution (200 μl) was added to the bottom of the transwell chamber (BIOFIL, 24-well plate, 8.0 μm). After gelation, the hydrogel was sterilized by UV. Subsequently, 100 μl of *S. aureus* suspension (10^5^ CFU/ml) was added to the transwell chamber and the chamber was placed on a well (24-well plate) containing 600 μl of the blank medium. The plates were cultured in a 37 °C incubator for 24 h, and the culture medium in the holes was diluted and counted.

### Wound healing evaluation

Kunming mice (~35 g) were anesthetized, and the back hair was removed. A circular wound with a diameter of 8 mm was prepared on the back of mice with a biopsy punch. Bacterial suspension (20 μl) (*S. aureus*, 10^8^ CFU/ml) was added to the wound, and 8-mm plate count agar covered with bacteria (100 μl, 10^8^ CFU/ml *S. aureus*, 37 °C culture 24 h) was placed on the wound. The wound was covered with a sterile closed PU (polyurethane) membrane for 24 h, and the agar was removed to obtain a bacterial infection wound model. Every 4 model mice were divided into a group, and the wounds were treated with (a) Gelatin hydrogel, (b) MepGel hydrogel, (c) MepGel hydrogel + NIR, and (d) MepGel microneedles (MepGelM) + NIR. One day after wound treatment, euthanasia of mice was performed by intraperitoneal injection of sodium pentobarbital (at a dose of 150 mg/kg). Before injection, the injection site was disinfected with alcohol. After injection, the reaction of the mice was closely observed to ensure the mice death (the mice stopped breathing, stopped heartbeat, dilated pupils, and did not respond to stimulation). Wound site tissue was taken, homogenized, diluted, and counted. The total bacterial count was calculated and divided by the wound area to get the bacterial count per square millimeter. The wound area was observed and photographed on the next 3, 6, 8, 12, and 16 d. On days 8 and 16, mice were sacrificed, and corresponding skin tissue samples were taken for Masson and hematoxylin and eosin (H&E) staining analysis. Quantitative statistical analysis of dermal thickness, inflammatory cells, collagen deposition, and hair follicles was also performed [24–26].

### Statistical analysis

One-way analysis of variance (ANOVA) followed by a Tukey multiple-comparison post hoc test for multiple comparisons with SPSS, version 24 (IBM) was conducted to determine the significant differences. *t* Test method was used for pairwise comparison. When *P* < 0.05, the data were considered statistically significant. *P < 0.05, **P < 0.01, and ***P < 0.001.

## Results

### Preparation and characterization of MepGel hydrogel

**Fig. 2.**
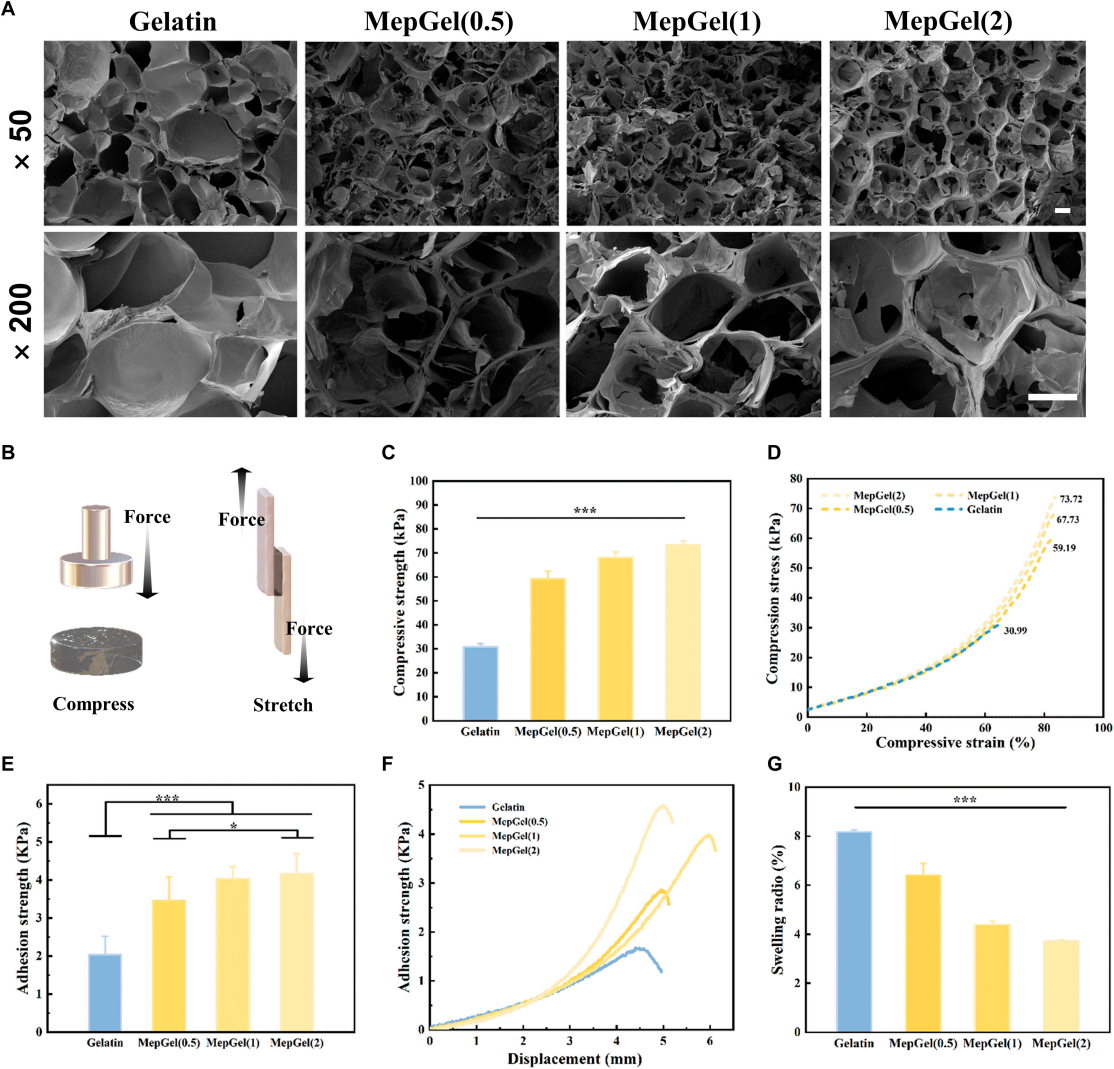
Morphology, mechanical properties, and swelling ratio of MepGel hydrogels. (A) SEM of hydrogels. Scale bar, 100 μm. (B) Schematic diagram of compression (left) and adhesion (right) test. (C) Compressive strength and (D) compression curves of hydrogels. (E) Adhesion strength and (F) stretching curves of pigskin (adhered by hydrogel). (G) Swelling ratio of hydrogels.*P < 0.05,**P < 0.01, ***P < 0.001.

MCBP was prepared according to previous reports. MCBP is mainly composed of glucose, galactose, and galacturonic acid, with a molecular weight of approximately 12.4 kDa (Fig. S1 and Table S1). Due to the abundant natural and planting resources of MCB, MCBP has a low cost and can be mass produced. A series of the MepGel hydrogel was prepared by heating and mixing gelatin solution with different contents of MCBP, and then annealing. The name of the MepGel hydrogel was determined by the MCBP contents, such as MepGel(1) representing an MCBP containing 1% (w/v). The effect of MCBP on the hydrogel network was evaluated by SEM. The results are shown in Fig. 2A. The interconnected porous structures in the hydrogel can be observed. With the addition of MCBP, the pore size decreased and the pore wall thickened obviously, which indicated that the mechanical properties of MCBP hydrogels may be improved [27]. Therefore, the mechanical properties of the MepGel hydrogel were evaluated by compression test (Fig. 2B, left). The results (Fig. 2C and D) showed that compared with the Gelatin hydrogel, the compressive strength of the MepGel hydrogel was significantly improved. The compressive strength of the MepGel hydrogel was about twice that of the Gelatin hydrogel, and its compressive strength increased significantly with the increase of MCBP concentration. The improvement of the mechanical properties of the composite hydrogel was related to MCBP.

In recent years, gelatin has been widely used as the hydrogel skeleton of biological adhesives [28]. One of the main reasons is that gelatin itself has tissue adhesion—a large number of amino groups on gelatin’s chain can react with aldehyde groups present on the skin through Schiff base reactions. The addition of MCBP can improve the mechanical properties of the composite hydrogels, which may lead to the improvement of the adhesive properties of this gelatin composite hydrogel. Therefore, we evaluated the adhesive properties of MepGel hydrogels by lap shear experiments (Fig. 2B, right). As shown in Fig. 2E and F, the addition of MCBP had a great impact on the adhesion performance of the composite hydrogel. The adhesive strength of MepGel hydrogels was about twice that of the Gelatin hydrogel. The improvement of adhesive properties is beneficial to wound closure and can effectively avoid the shedding of wound dressings during sports [29]. The water absorption of hydrogel will cause its volume change. When used as a wound sealant, the swollen hydrogel may compress the wound, which is not conducive to wound healing [30]. Therefore, the swelling performance of the MepGel hydrogel was evaluated. The results (Fig. 2G) showed that compared with the Gelatin hydrogel, the MepGel hydrogel had a lower swelling ratio. Moreover, with the increase of MCBP concentration, the swelling ratio of the MepGel hydrogel gradually decreased. This suggested that there was an interaction between MCBP and gelatin, which increased hydrogel crosslinking density.

### Rheology and plasticity of MepGel hydrogel

MCBP has unique rheological properties and is widely used as a stabilizer and coagulant aid in food hydrogels [31]. Li and colleagues [17] found that the addition of MCBP significantly altered the rheological behavior of cassava starch. The interaction between MCBP and cassava starch is mainly hydrogen bonding, which leads to the thermal reversibility of the composite hydrogel. Moreover, studies have shown that the addition of polysaccharides can effectively improve the thermal stability of the Gelatin hydrogel [32]. Therefore, we evaluated the rheological effect of MCBP on the composite hydrogel.

**Fig. 3.**
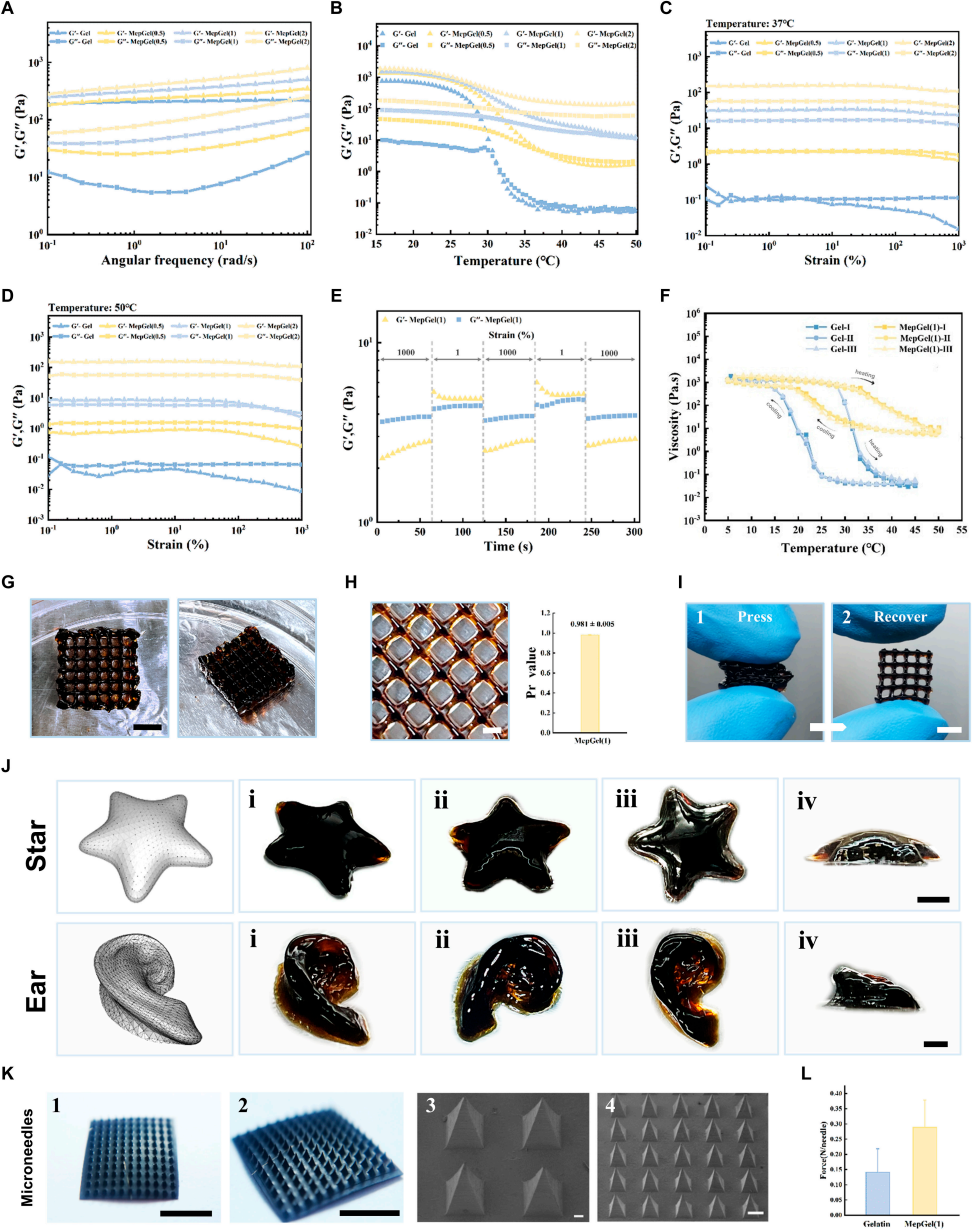
Rheology and plasticity evaluation of MepGel hydrogels. (A to F) Rheological study of hydrogels. (A) Oscillating frequency experiment. (B) Temperature sweep experiment. (C) Oscillation amplitude sweep tests at 37 °C and (D) 50 °C. (E) Alternating oscillatory time sweep test. (F) Flow temperature ramp test. (G) Matrix printed with the MepGel(1) hydrogel ink. Scale bar, 6 mm. (H) Study on the fidelity of the MepGel(1) ink. Scale bar, 1.5 mm. (I) Anti-deforming ability of MepGel(1)-printed structure. Scale bar, 3 mm. (J) Printed complex structures with the MepGel(1) ink. Top, star; bottom, ear. Scale bar, 5 mm. (K) Morphology of MepGel(1) microneedles. Scale bar, 4 mm (1 and 2), 100 μm (3), and 300 μm (4). (L) Compression test of microneedles.

The oscillation frequency experiment showed that the storage modulus (*G*′) of all hydrogels was greater than the loss modulus (*G*″) at 25 °C, 10^−1^ to 10^2^ angular frequency (Fig. 3A). These hydrogels all exhibited viscoelastic behavior similar to that of soft tissue. They belong to viscoelastic solids; *G*′ was about 10 times *G*″, at 1 Hz (≈6.28 rad/s) [33]. It is easy to see that the addition of MCBP improved *G*′ and *G*″ of the composite hydrogel, suggesting that MCBP can optimize the mechanical properties of the hydrogel, which was consistent with the results of compression experiments. Next, we investigated the effect of MCBP on the phase transition temperature of the Gelatin hydrogel (Fig. 3B). The results showed that *G*′ and *G*″ of the Gelatin hydrogel approached gradually with the increase of temperature; *G*′ = *G*″ occurred at about 31 °C, moduli at 1 Hz. The Gelatin hydrogel then transformed from an elastic solid into a liquid. The temperature–modulus curve of the MepGel(0.5) hydrogel was similar to that of the Gelatin hydrogel, but the hysteresis of phase transition temperature can be observed; *G*′ < *G*″ occurred after 37 °C. On the contrary, no phase transition (*G*′ = *G*″) was observed in MepGel(1) and MepGel(2) hydrogels as the temperature increased to 50 °C. Interestingly, when the temperature rose to 50 °C, *G*′ of the MepGel(1) hydrogel was close to *G*″ and showed a certain fluidity, which we called an SF state.

Considering that wound dressings need to be stable at body temperature, we carried out oscillatory amplitude sweep measurements on the MepGel hydrogel in the shear strain range of 0.1% to 1,000% at 37 °C. As shown in Fig. 3C, Gelatin and MepGel(0.5) hydrogels showed lower modulus at 37 °C and exhibited fluid properties at high shear strain (*G*′ < *G*″). On the contrary, MepGel(1) and MepGel(2) hydrogels exhibited a stable gelation state at 37 °C, and *G*′ was always greater than *G*″ in the range of shear strain. Since we envisaged changing the state of the hydrogel by temperature, we assessed the rheological behavior of the MepGel hydrogel at 50 °C—short periods of exposure to that temperature would not cause damage to normal tissues [34]. As shown in Fig. 3D, at this temperature, Gelatin and MepGel(0.5) hydrogels were liquid, while the MepGel(2) hydrogel continued to remain in an SS state. Interestingly, at low shear strain, *G*′ of the MepGel(1) hydrogel was very close to *G*″, which was consistent with the results of the oscillation frequency experiment. However, with the increase of shear strain, *G*″ of the MepGel(1) hydrogel was gradually larger than *G*′, showing pseudoplasticity. The strain amplitude sweep results of MepGel(1) at 50 °C showed that *G*′ decreased rapidly in the critical strain region (γ = 1,000%) and was significantly smaller than *G*″ (Fig. 3E). This indicated that the hydrogel network collapsed and loosened under large amplitude force. However, when the amplitude returned to γ = 1%, the modulus also recovered rapidly, indicating that the hydrogel network returned to the original state. The above changes mean that the MepGel(1) hydrogel is injectable at 50 °C.

Thermosensitive hydrogels usually have strong plasticity. However, the state reversibility and stability of thermosensitive hydrogels are poor and may be affected by the previous state [35]. Therefore, we repeatedly raised and lowered the temperature of the MepGel(1) hydrogel to observe the changes in its viscosity. The results (Fig. 3F) showed that similar to most thermosensitive hydrogels, the viscosity curves of hydrogel were difficult to overlap in the process of heating/cooling. However, the area surrounded by the viscosity curve of the MepGel(1) hydrogel was almost identical during the triple heating and cooling cycle, indicating that it can effectively avoid the influence of the previous state. Interestingly, the viscosity of the Gelatin hydrogel varied across 5 orders of magnitude over a temperature range of 5 to 50 °C, while that of the MepGel(1) hydrogel remained about 2 orders of magnitude. The high viscosity change of thermosensitive hydrogels is unfavorable to their extrusion molding. Especially for the Gelatin hydrogel, the receiver needs a low temperature to achieve fast gelatin time, which often leads to syringe blockage. In contrast, the viscosity of the MepGel(1) hydrogel varied a little from 50 °C to room temperature (25 °C), and its extrusion molding was temperature independent.

The results of rheology indicated that the MepGel(1) hydrogel had high plasticity. We expect that it can be easily shaped to seal wounds of various shapes. Therefore, we tried to use extrusion 3D printing and reverse molding to prepare the MepGel(1) hydrogel into various shapes. Figure 3G shows a matrix (12 × 12 × 3 mm) printed using the MepGel(1) hydrogel as an ink for the first time. The matrix reflected the high fidelity and resolution of the MepGel(1) hydrogel ink. The Pr value is a key parameter for evaluating the fidelity of printed materials, and a value close to 1.0 indicates a higher fidelity of the printed material [36]. We further evaluated the fidelity of the MepGel(1) hydrogel as the printing ink by the Pr value. The results showed that the Pr value of the MepGel(1) hydrogel ink was 0.981 ± 0.005 (Fig. 3H, right), which was superior to the Gelatin hydrogel (Pr = 0.943 ± 0.006; Fig. S2, right). It was observed that compared to the printing lines with uneven thickness in the Gelatin ink, the lines of the MepGel(1) ink were smoother and more uniform (Fig. 3H, left, and Fig. S2, left). Interestingly, because of the excellent mechanical properties of the MepGel(1) hydrogel, the printed model showed robustness. After repeated presses, the model can still recover to its original appearance (Fig. 3I). Next, we used the MepGel(1) hydrogel to print complex structures such as stars and ears (Fig. 3J). The printed structure was consistent with the computer model and excellently supported its gravity (especially the concave contour of the ear). The enormous printing potential of the MepGel(1) hydrogel enabled it to easily prepare various 3D models to meet the closure needs of various shapes of wounds. Importantly, in situ hydrogels or injectable hydrogels used for wound closure commonly have problems such as raw material leakage, dilution, and unstable mechanical properties [3]. Therefore, the MepGel(1) hydrogel has shown particular charm in easily preparing custom dressings with excellent mechanical properties for wound closure.

In addition, we tried to prepare the MepGel(1) hydrogel microneedles by traditional micro-forming technology. The results showed that the MepGel(1) hydrogel in the SF state could be processed into a micrometer structure (Fig. 3K). The microneedle patch consisted of 11 × 11 microneedle arrays. Each microneedle was presented as a pyramid, with a height of 800 μm and a base width of 350 μm. The compression experiment (Fig. 3L) showed that when pressed down to 600 μm, the compression force of the MepGel(1) microneedle per needle was about 0.3 N, which was better than the Gelatin microneedle (0.15 N) and significantly exceeded the minimum force required to penetrate the stratum corneum (0.045 N) as previously reported [37]. This fine micrometer-scale array has the potential to puncture bacterial biofilms and increase the antibacterial efficiency of dressings [38].

Above all, the MepGel(1) hydrogel showed excellent mechanical properties, and it was possible to give the MepGel(1) hydrogel different shapes by 3D printing, and likewise to prepare it into microneedles by centrifugal casting method. The results highlight that the MepGel(1) hydrogel is highly malleable and can be customized to match different sites, shapes, and depths of wounds to meet the needs of various wounds. The MepGel(1) hydrogel also avoids the problems of poor therapeutic effect caused by leakage of raw materials, dilution, and unstable mechanical properties that are usually associated with some in situ or injectable hydrogels, while this MepGel(1) hydrogel is able to take care of wounds and promote wound healing in a more effective way.

### Hydrogen bond cross-linking and self-healing ability

**Fig. 4.**
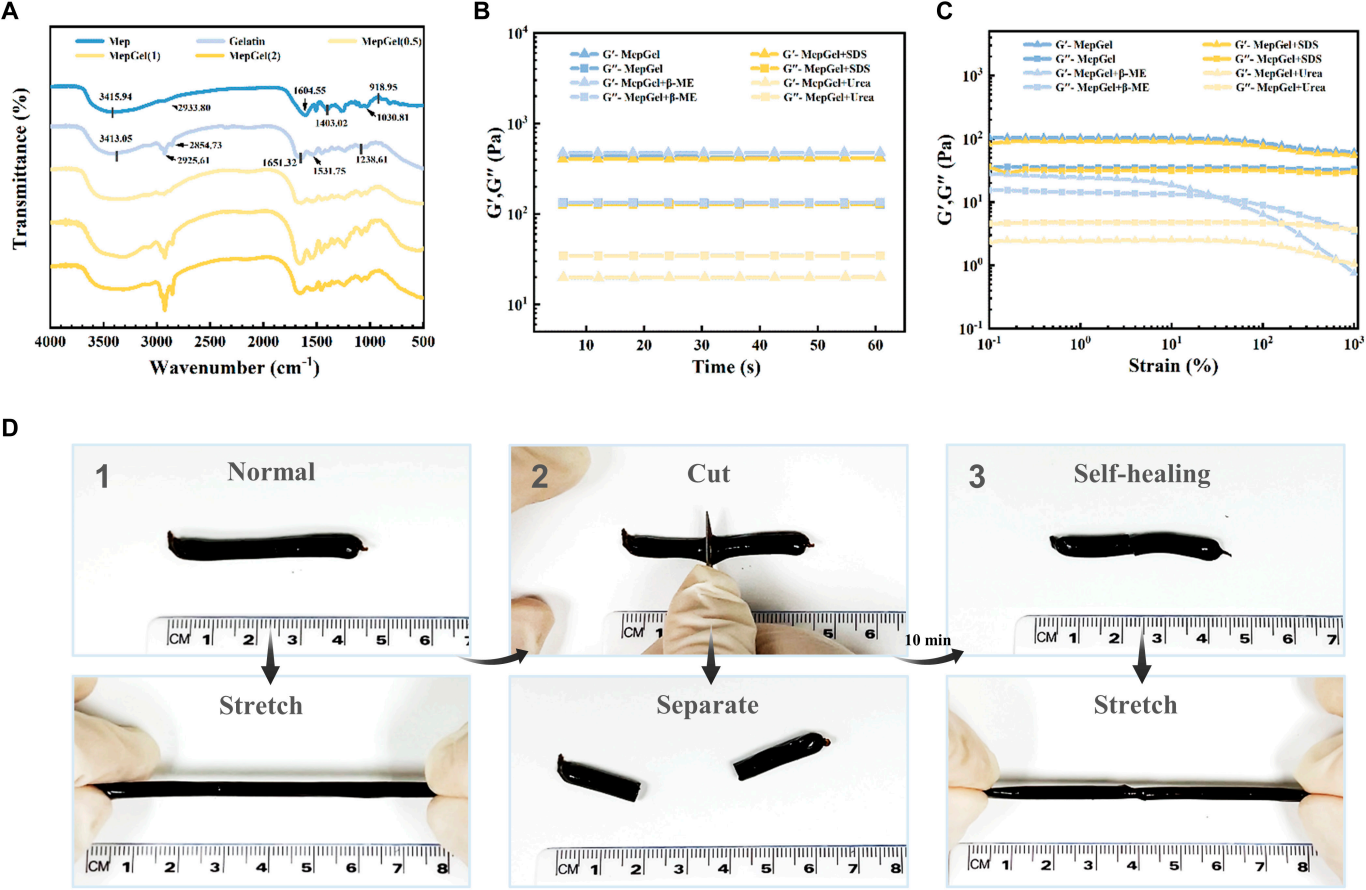
H-bonding network of the MepGel hydrogel and its self-healing properties. (A) Infrared spectra of hydrogels. (B) Oscillation time scanning test of the samples at 25 °C. (C) Oscillatory amplitude scan test of the samples at 37 °C. (D) Study on the self-healing property of the MepGel(1) hydrogel.

The addition of MCBP significantly changed the physical properties of the composite hydrogel, prompting us to further investigate the interaction between the polysaccharide and protein. We first used FTIR to observe whether there were new chemical bonds formed between MCBP and gelatin. As shown in Fig. 4A, MCBP presented a typical spectrum of polysaccharides. The values 3,415.94 and 2,933.8 cm^−1^ were attributed to the stretching vibrations of O–H and saturated C–H bonds, respectively. The absorption peaks of 1,604.55 and 1,403.02 cm^−1^ were attributed to the stretching vibrations of carbonyl carbon–oxygen double bonds and single bonds in uronic acid. The values 800 to 1,200 cm^−1^ belong to the fingerprint region of polysaccharides, where the absorption peak at 1,030.81 cm^−1^ was attributed to C–O–C stretching and 918.95 cm^−1^ was generated by glycosidic connections. Moreover, the absorption peak of gelatin was consistent with reports; the characteristic absorption bands of amide I, amide II, and amide III appeared at 1,651.32, 1,531.75, and 1,238.61 cm^−1^, respectively [39]. Compared with the 2 raw materials, no new absorption peaks were found in the spectra of MepGel hydrogels, indicating that no new chemical bonds were formed in the hydrogel network. However, with the addition of MCBP, the absorption peak at around 3,400 cm^−1^ attributed to O–H showed a red shift and widened, indicating potential hydrogen bonding interactions [40].

To further study the intermolecular interaction of the MepGel hydrogel, we added sodium dodecyl sulfate (SDS), urea, and β-ME to the pre-hydrogel solution, respectively, to destroy hydrophobic interaction (SDS), hydrogen bonding (urea), and disulfide bonding [β-mercaptoethanol (β-ME)] in the hydrogel. The changes in mechanical properties of hydrogels caused by the addition of these reagents were observed by the rheometer. At 25 °C, the addition of urea significantly changed the rheological curve of the MepGel hydrogel at a constant angular frequency of 100 rad/s and showed the rheological properties of the liquid (*G*′ < *G*″). However, the addition of SDS and β-ME did not seem to affect the mechanical properties of the hydrogels, and their rheological curves after addition almost overlapped those of the MepGel(1) hydrogels (Fig. 4B). Next, the oscillatory amplitude scan measurements were conducted at 37 °C (Fig. 4C). The addition of SDS did not change the rheological properties of the MepGel hydrogel. At 37 °C, the rheological properties of the MepGel + SDS hydrogel group were consistent with those of the MepGel hydrogel group in the shear strain range. In contrast, the addition of β-ME changed the steady state of the MepGel hydrogel at 37 °C, decreased the modulus of the hydrogel, and exhibited the characteristics of shear thinning. Therefore, we believe that there was mainly hydrogen bond interaction between MCBP and gelatin molecular chain in MepGel hydrogels. Second, the disulfide bond also plays a role in the formation of the MepGel hydrogel.

The MepGel hydrogel was based on a hydrogen bond network, indicating that it has a self-healing property [41]. As shown in Fig. 4D (Movie S1), the cylindrical MepGel(1) hydrogel (diameter: 0.4 cm) could be stretched to twice the original length before being cut. Subsequently, the hydrogel was cut into 2 segments, and the 2 segments were spliced and placed. After 10 min, we stretched this MepGel(1) hydrogel again. As a result, after being stretched to one time of its length, the hydrogel remained intact without breaking along the incision. The MepGel(1) hydrogel’s excellent self-healing properties result in an extended service life as a wound dressing, preventing exposure of the wound due to damaged dressing [42].

### NIR stimulus response of MepGel hydrogel

**Fig. 5.**
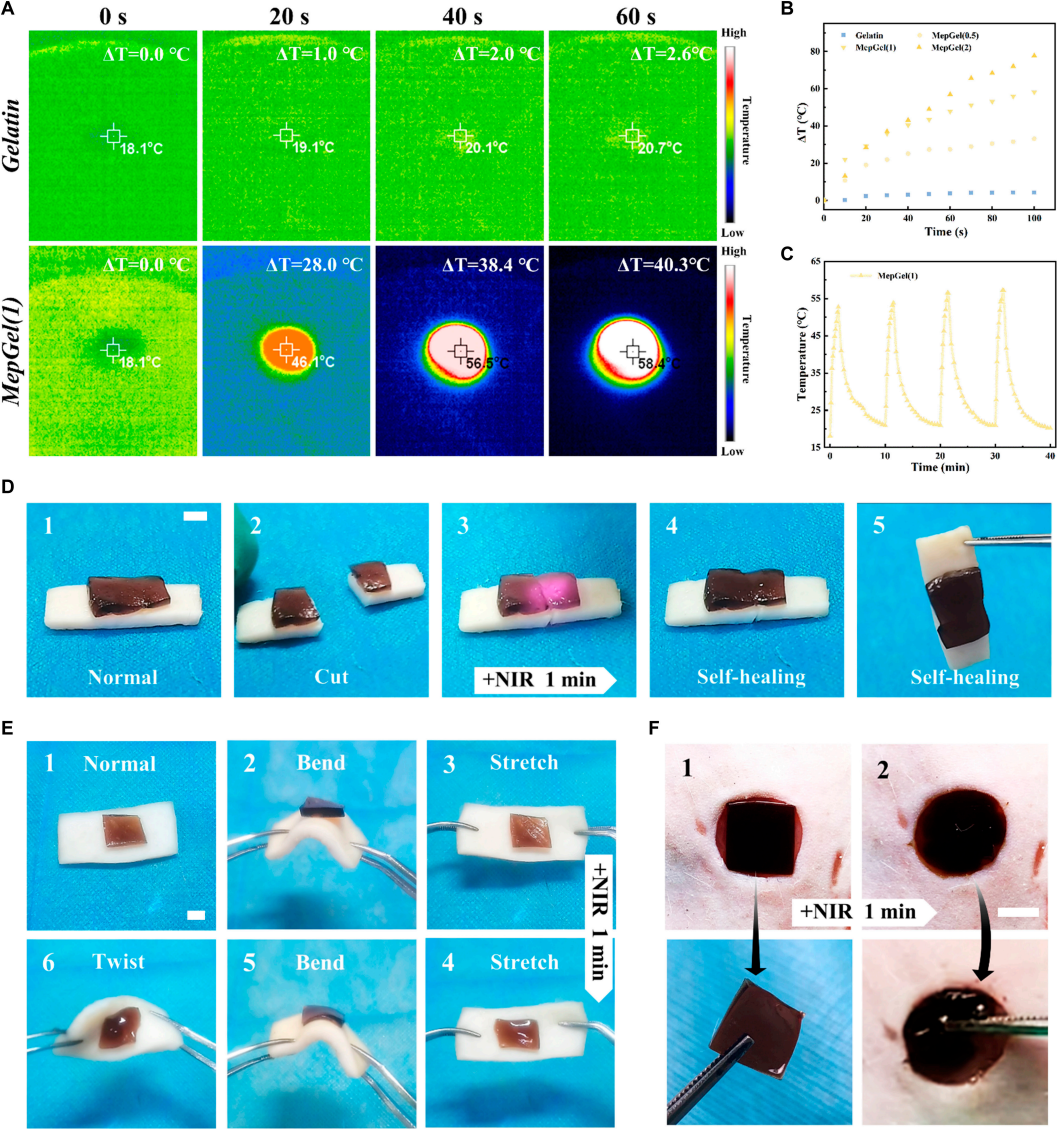
NIR stimulus response of the MepGel hydrogel. (A) Infrared thermography of hydrogels. (B) Photothermal heating curves of samples. (C) Temperature variation of the MepGel hydrogel with repeated NIR irradiation. (D) The NIR PTT (photothermal therapy) promotes hydrogel self-healing. (E) The NIR PTT helped the recovery of the hydrogel’s adhesion. (F) The NIR PTT made hydrogel further fit the wound. Scale bar, 4 mm.

The absorption of MCBP in the UV and NIR region indicated that the MepGel hydrogel may have a potential NIR photothermal effect (Fig. S3) [43]. The photothermal effect was evaluated by irradiating hydrogel samples with a NIR laser (2 W/cm^2^) for 1 min (Fig. 5A and B). The composite hydrogel had a photothermal effect with the addition of MCBP, and the increase in MCBP concentration led to the more obvious NIR photothermal effect. The temperature of the Gelatin hydrogel (ΔT ≈ 3.8 °C) did not change significantly after irradiation for 60 s. On the contrary, the temperatures of MepGel(0.5), MepGel(1), and MepGel(2) hydrogels increased by 27.4, 47.9, and 56.9 °C, respectively, after 60-s irradiation. In addition, the repeatable photothermal properties of MepGel hydrogels were evaluated by repeated irradiation heating and natural cooling (Fig. 5C). After 4 heating and cooling cycles, the MepGel hydrogel still maintained excellent photothermal performance.

The MepGel(1) hydrogel exhibits significant NIR stimulus response due to the photothermal effect and hydrogen bonding network, expanding its functionality. NIR irradiation led to the rapid self-healing of the MepGel(1) hydrogel. As shown in Fig. 5D, the fresh pigskin and the attached MepGel(1) hydrogel were cut at the same time. Then, after 1 min of NIR radiation, the hydrogel self-healed and successfully connected the whole pigskin. During this process, NIR irradiation led to an increase in the temperature of the hydrogel and produced a large number of free acidic hydrogen atoms and Y atoms (O, N) with high electron density. With the end of irradiation, cooling led to hydrogen bond recombination, and the hydrogel eventually showed rapid self-healing. In addition, the experiments showed that the NIR radiation was helpful to the recovery of the adhesion of the MepGel(1) hydrogel (Fig. 5E). After NIR irradiation, the hydrogel was attached to the pigskin. As the pigskin was twisted, bent, or stretched, the hydrogel also presented a corresponding stress state. Due to the existence of a hydration layer and surface dust, the long-term storage of hydrogels often leads to adhesion failure. After the MepGel(1) hydrogel was heated up by NIR radiation, the original hydrogen bond hydrogel network collapsed, the hydrogel became “soft”, and the fluidity increased, leading to more sufficient interface contact, and finally achieved adhesion. Finally, NIR radiation could make the MepGel(1) hydrogel further fit the shape of the wound. As shown in Fig. 5F (Movie S2), after NIR irradiation, the square MepGel(1) hydrogel sealed the round wound. This also depended on the hydrogen bond network fracture caused by the photothermal effect heating, and the hydrogel changed from a hard “SS” state to an easy to manufacture “SF” state. Subsequently, the MepGel(1) hydrogel flowed under the action of external force and filled the whole skin defect. The MepGel(1) hydrogel showed various NIR photothermal responses, which enabled it to overcome the nursing challenges of wound adhesion and closure.

### Antibacterial effect of MepGel hydrogel

**Fig. 6.**
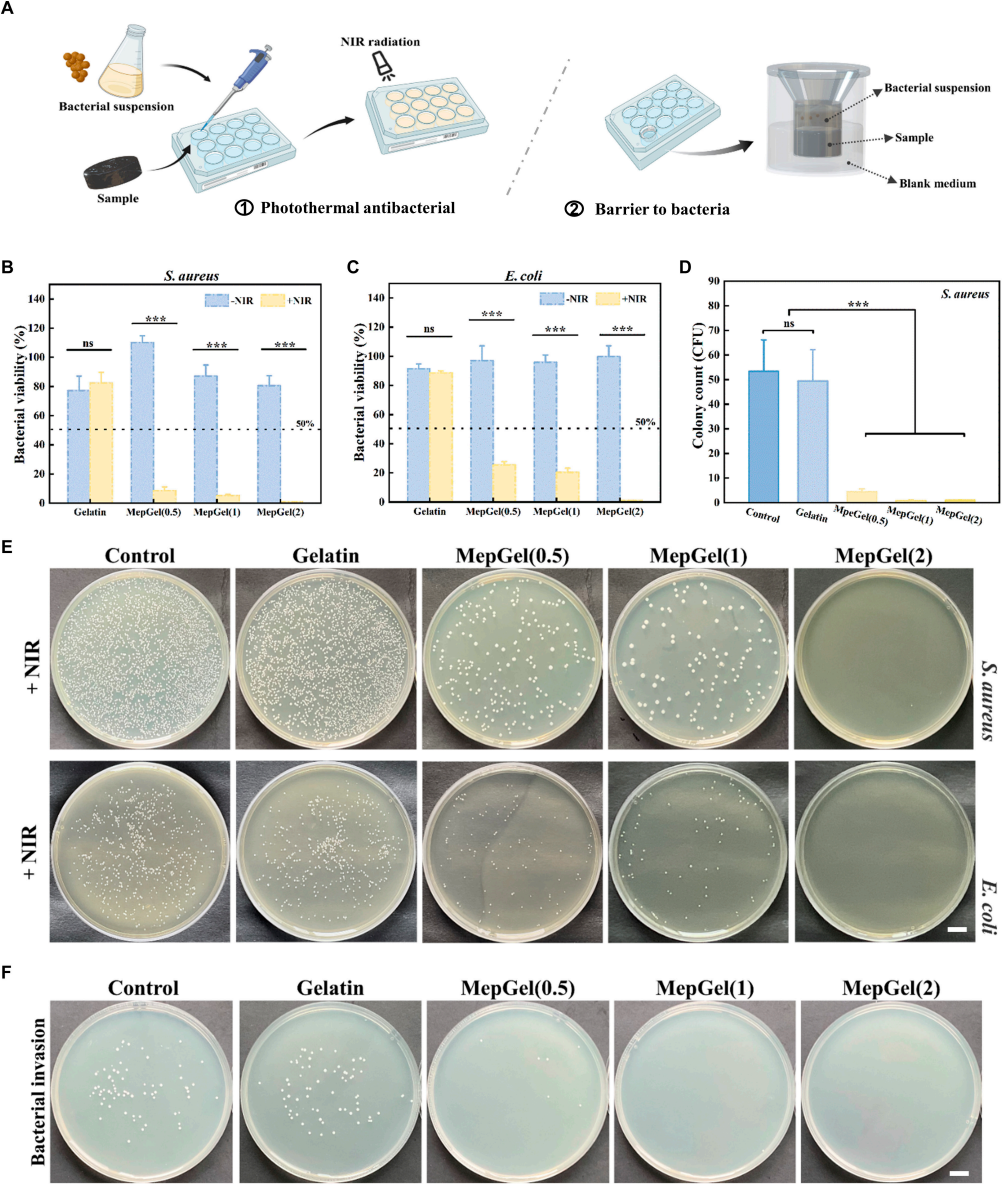
Antibacterial effect of the MepGel hydrogel. (A) Schematic diagram of the photothermal antibacterial test (left) and bacterial invasion test (right) of hydrogels. (B) Bacterial activity of *S. aureus* and (C) *E. coli* cocultured with samples. (D) Bacterial invasion test of hydrogels. (E) Representative colony agar plate images after + NIR treatment. (F) Representative images of colony agar in bacteria invasion test. Scale bar,1 cm. *P < 0.05,**P < 0.01, ***P < 0.001.

The photothermal effect of the MepGel hydrogel also indicated that it can cause physical damage to bacteria by heating. Therefore, the antibacterial properties of the MepGel hydrogel were investigated by in vitro antibacterial experiments (Fig. 6A, left). As shown in Fig. 6B and C, in the antibacterial experiment with *S. aureus* as the G+ bacteria model and *E. coli* as the G− bacteria model, the antibacterial effect of the Gelatin hydrogel against both bacteria was not obvious. The presence/absence of NIR irradiation did not affect the antibacterial effect of the Gelatin hydrogel. However, the antibacterial property of the MepGel hydrogel changed significantly under the condition of NIR irradiation. The bacterial activity of the MepGel + NIR group remained below 25%. Among them, the antibacterial rate of the MepGel(2) group to both bacteria reached 99% in a limited irradiation time. Through the agar plate, we can observe the difference in the antibacterial effect of different groups with or without NIR irradiation (Fig. 6E and Fig. S4). Importantly, by adjusting the intensity and irradiation time of NIR light, the antibacterial effect of photothermal materials can be enhanced [44,45]. These methods can also prevent skin burns caused by excessive heating.

The damage to the skin barrier made the exposed tissues face the risk of bacterial infection at any time. The wound dressing should cover the wound while providing a barrier for the wound to prevent bacterial invasion. The network structure of the hydrogel provided a good filter screen for blocking bacteria [46]. Therefore, we evaluated the blocking effect of the MepGel hydrogel on bacteria through bacterial invasion experiments (Fig. 6A, right). As shown in Fig. 6D and H, the Gelatin hydrogel had little effect in preventing bacterial invasion due to its complete melting into a liquid at 37 °C. In contrast, the MepGel hydrogel showed a protective effect against bacterial invasion. This was due to MCBP reinforcing the Gelatin hydrogel network through hydrogen bonding, resulting in a higher phase transition temperature of the MepGel hydrogel. It was conceivable that the MepGel(1) hydrogel can be easily prepared into wound shape, covered, and adhered to the wound through NIR radiation to better protect the wound from external bacteria.

### In vitro cell experiments of MepGel hydrogel

**Fig. 7.**
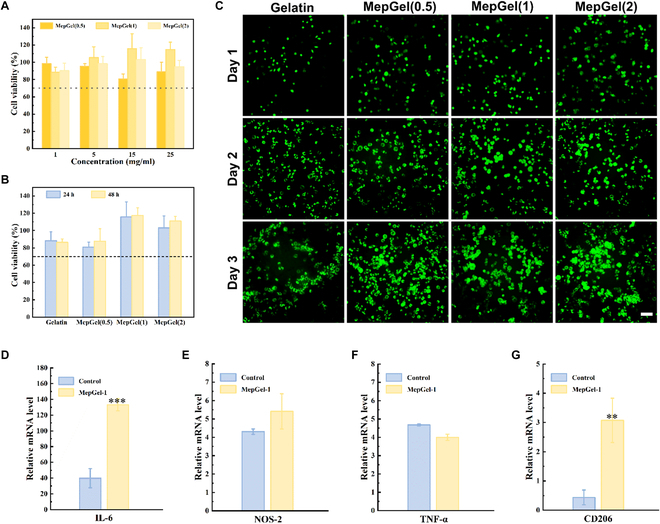
The cytotoxicity and macrophage stimulation of the MepGel hydrogel. (A) Cell viability of L929 after 24 h and (B) 48 h of culture with the hydrogel medium. (C) Cell staining analysis of L929 directly cultured on the hydrogel. Scale bar, 200 μm. (D to G) Real-time PCR analysis of Raw 264.7 gene expression after 24 h of coculture with hydrogel. *P < 0.05,**P < 0.01, ***P < 0.001.

Good biocompatibility is the basis of biomaterial research [47]. The cytotoxicity of the MepGel hydrogel was assessed by using L929 cells as a model. First, we used a hydrogel coculture medium to culture cells. The results are shown in Fig. 7A and B. After 24-h culture in different concentrations of the hydrogel coculture medium, the viability of cells in each group was more than 70%, suggesting that the material had no obvious cytotoxicity. Moreover, after 48 h of continuous culture, the cells still maintained good cell viability. The cells were cultured in direct contact with the hydrogel to further investigate the biocompatibility of the MepGel hydrogel. As shown in Fig. 7C, live cells were stained with calcein-AM and displayed green fluorescence under a fluorescence microscope. The cells in each group were oblate or fusiform and showed stable proliferation.

Almost all biomaterials can induce foreign-body reactions in different degrees after direct contact with tissues [48]. These reactions may promote tissue repair or lead to failure in wound healing. Macrophages were involved in almost all stages of foreign-body response. Therefore, we cultured Raw 264.7 cells on the MepGel(1) hydrogel to further observe the stimulatory effect of this hydrogel on macrophages. Here, we use gelatin/alginate/Ca^2+^ interpenetrating network hydrogel as a control. As an inert block copolymer, sodium alginate was considered to cause mild foreign-body reactions [49]. Real-time PCR data were similar to previous reports. MCBP-induced macrophages express more genes related to the clearance of pathogenic microorganisms (Fig. 7D to F). However, this does not mean that the MepGel(1) hydrogel will cause persistent inflammation. We found a significant increase in mRNA levels of the anti-inflammatory M2 phenotype-related marker CD206 (Fig. 7G). This may be attributed to a significant increase in the secretion of interleukin-6 (IL-6), a dual functional factor in the immune system, which induces macrophage polarization toward the M2 phenotype [50]. Therefore, the MepGel(1) hydrogel may promote the elimination of pathogens by regulating immunity and accelerating the process of wound healing.

### Promote bacterial infected wound healing

**Fig. 8.**
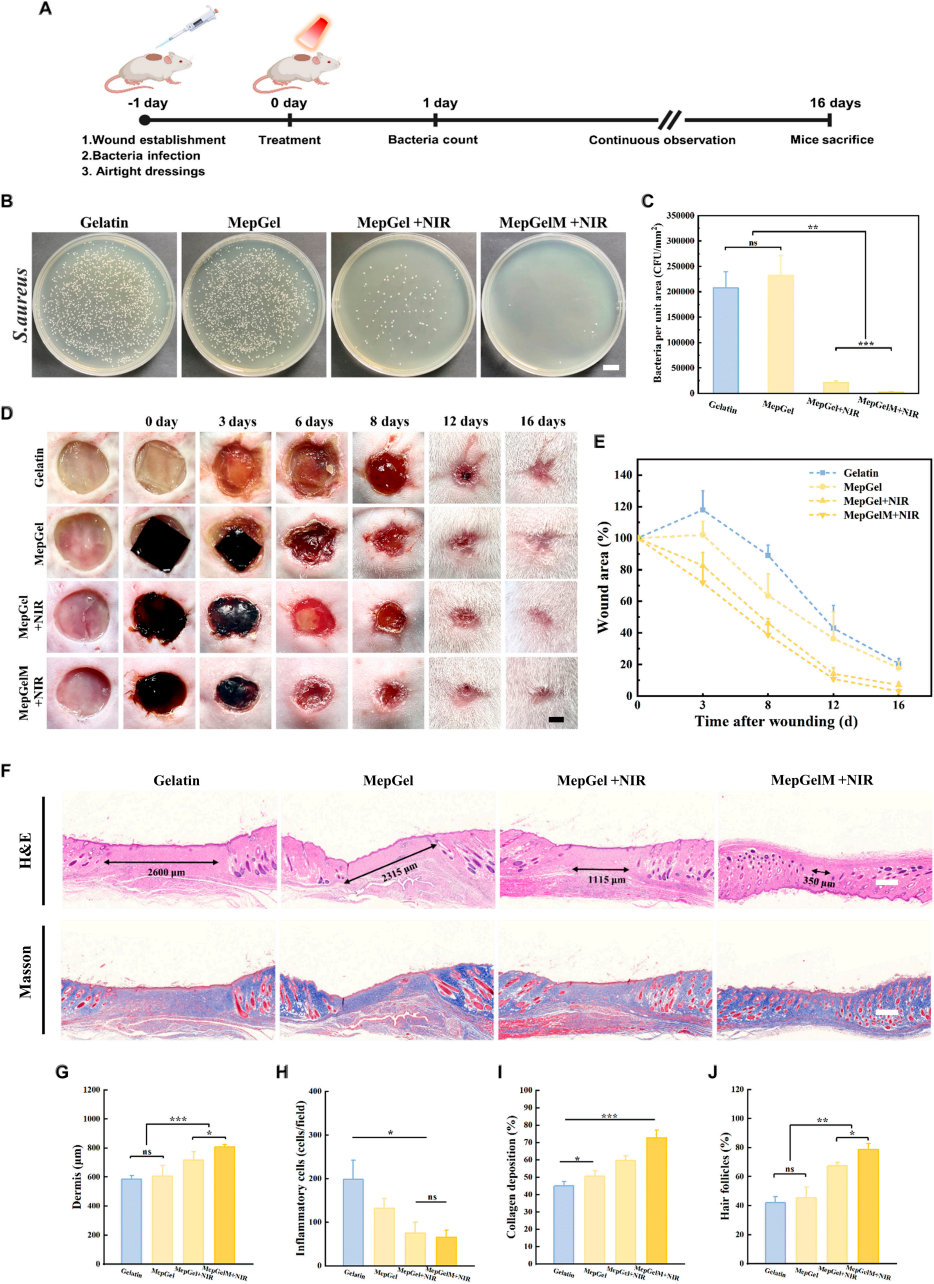
In vivo performance of the MepGel hydrogel in healing infected wound. (A) Schematic diagram of in vivo infected wound healing experiment. (B) Representative agar plate images of antibacterial test. Scale bar, 1 cm. (C) Number of bacteria per square millimeter wound. (D) Representative images of wound healing with different treatments. Scale bar, 2 mm. (E) Statistical curve of the wound area. (F) H&E- and Masson-stained images of wounds from different treatment groups on day 16. Scale bar, 500 μm. (G) Wound dermal thickness in MepGelM + NIR, MepGel + NIR, MepGel, and Gelatin groups at day 16. (H) Inflammatory cells in skin tissue in the MepGelM + NIR, MepGel + NIR, MepGel, and Gelatin groups on day 16. (I) Collagen deposition in skin tissue of MepGelM + NIR, MepGel + NIR, MepGel, and Gelatin groups on day 16. (J) Hair follicles in skin tissue of MepGelM + NIR, MepGel + NIR, MepGel, and Gelatin groups on day 16. *P < 0.05, **P < 0.01, ***P < 0.001.

Finally, we developed a mouse model of wounds with bacterial infection to evaluate the antibacterial and wound healing-promoting effects of the MepGel hydrogel in vivo. The experimental process was shown in Fig. 8A. A circular wound with a diameter of 8 mm was made on the back of the mouse. Bacterial suspensions and colony-covered agar media were added to the wound site, and the wound was sealed with a sterile PU membrane. After 24 h, the agar medium at the wound site was removed and the wound was treated. As shown in Fig. 8B and C, the wound tissue treated with Gelatin or MepGel hydrogel alone was abundant in bacteria. However, the wound infection in the MepGel + NIR group was significantly improved, and the number of bacteria per square millimeter in the wound site was about ^1^/_10_ of that in the Gelatin and MepGel groups. Interestingly, there was a significant difference in treatment with the MepGel hydrogel or microneedles under NIR irradiation conditions. Microneedles showed a better effect than did the hydrogel therapy. This may be because the microneedles can pierce the bacterial biofilm and conduct heat conduction more efficiently. Figure 8D and E shows that the wound surface was covered with a milky translucent biofilm before treatment. Severe wound infection was observed on days 3 and 6 in both Gelatin and MepGel groups. The wound was inflamed and increased in size. In addition, wounds in both groups showed a significant delay in healing, with the site of the wound still clearly visible at 16 d. In contrast, there was no apparent skin redness during wound healing in the NIR irradiated MepGel hydrogel as well as in the MepGel microneedles group, and showed minimal wound area on day 16.

H&E staining and Masson staining were used to further evaluate wound healing. As shown in Fig. 8F (Fig. S5), after 8 d of treatment, the skin gaps in the Gelatin and MepGel groups were vaguely visible, while the skin gaps in the MepGel + NIR group and the MepGelM + NIR group were filled with new granulation tissue. On the 16th day, the skin epidermis structure of all groups was intact, but the wounds treated by the Gelatin and MepGel groups could see a large area of granulation tissue with inflammatory infiltration. However, this situation was significantly improved in the MepGel + NIR group and the MepGelM + NIR group. Especially for the wounds treated with MepGelM + NIR, multiple hair follicles and sebaceous glands can be seen in the dermis. In addition, Masson staining showed that the wound treated with MepGelM + NIR showed the densest and thickest collagen fibers parallel to the wound. Wound healing is a complex and sequential physiological process that includes hemostasis, inflammation, migration, proliferation, and remodeling. The results showed that the dermal thickness on day 16 in the MepGelM + NIR group was much higher than that in the MepGel + NIR group, the MepGel group, and the Gelatin group (Fig. 8G). On the 16th day, the inflammatory response of the MepGelM + NIR group was much lower than that of the other 3 groups, and only a small number of inflammatory cells could be found. The order of inflammatory response was MepGelM + NIR < MepGel + NIR < MepGel < Gelatin, which was opposite to the wound healing performance in vivo, indicating that inflammation had a greater impact on wound healing. Moreover, the composite hydrogel material treated by NIR irradiation can effectively reduce inflammation (Fig. 8H). In addition, compared with the MepGel and Gelatin groups, the MepGelM + NIR group and the MepGel + NIR group had more collagen deposition (Fig. 8I). Meanwhile, the MepGelM + NIR group showed a large number of regenerated hair follicles in the wound center, and the MepGel + NIR group showed a slightly lower number of hair follicles, while the other 2 groups showed limited hair follicle regeneration (Fig. 8J). Regeneration of skin appendages such as hair follicles helps to improve the quality of healing. The results suggested that MepGel, a novel photothermal material, effectively improved bacterial infections and promoted wound healing.

## Conclusion

In summary, a low-cost, ecofriendly, natural hydrogel wound dressing was successfully prepared with versatile properties. The MepGel hydrogel was highly malleable and could be easily shaped to provide better closure of wounds. MepGel hydrogels possessed remarkable mechanical properties, self-healing abilities, and biocompatibility and can effectively resist bacterial invasion. Importantly, the hydrogel has a NIR thermal effect that provided antibacterial properties and benefited and promoted wound healing by re-adhesion, further covering the wound and accelerating self-healing of dressings. Therefore, the hydrogel effectively overcomes the common problems of material leakage and poor mechanical strength of injectable adhesive dressings. The hydrogel could meet the needs of daily wound care and showed great potential in clinical application.

## Data Availability

The datasets used and/or analyzed during the current study are available from the corresponding author on reasonable request.
