# Supplementary material for: H-Bonds Enhanced Natural Polyphenols Bined Polysaccharide/Gelatin Composites with Controlled Photothermal Stimulation Phase Transition for Wound Care
Source: Biomater Res. 2024 Sep 13;28:0082. doi: 10.34133/bmr.0082 (PMC11395704; doi:10.34133/bmr.0082)
Supplement: Supplementary 1 — Supplementary Text Figs. S1 to S5 Table S1 Movies S1 and S2 [file bmr.0082.f1.zip › Supporting Information.docx]

Supporting Information

# Experimental section

## Chemical properties of MCBP

The contents of total sugar and protein in MCBP were determined by phenol-sulfuric acid and bicinchonininc acid method. The monosaccharide composition in MCBP was determined by ion chromatography (IC). Briefly, 5 mg of MCBP was added to 2 mL 3 M TFA, hydrolyzed at 120 ℃ for 3 h. Transferred the hydrolysis solution to a tube and blow dry with nitrogen. Added 5 mL of deionized water and mix well. Took 50 μL and added 950 μL of deionized water. Centrifuged at 12000 rpm for 5 min and took supernatant for IC analysis. In addition, the molecular weight of polysaccharide was determined by HPGPC. Briefly, 2 mg MCBP was dissolved in 1 ml of mobile phase solution. After centrifugation, the supernatant was extracted, filtered with 0.22 μm aqueous microporous membrane, and then the sample was transferred to 1.8 mL injection vial for testing.

# Results


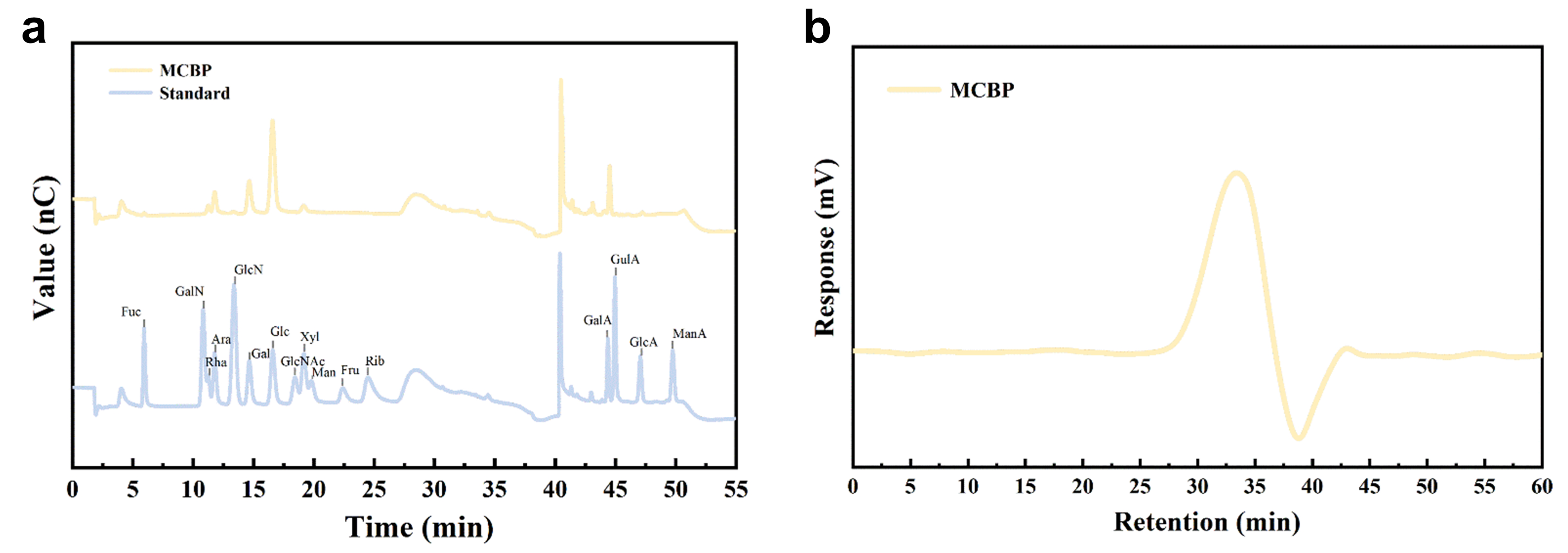


**Fig.S1.** The monosaccharide composition and molecular weight of MCBP. a)  ion chromatogram of MCBP and monosaccharide standards. b) HPGPC spectrum of MCBP.

**

Fig.S2.** Study on the fidelity of Gelatin ink. Scale bar: 1.5 mm.
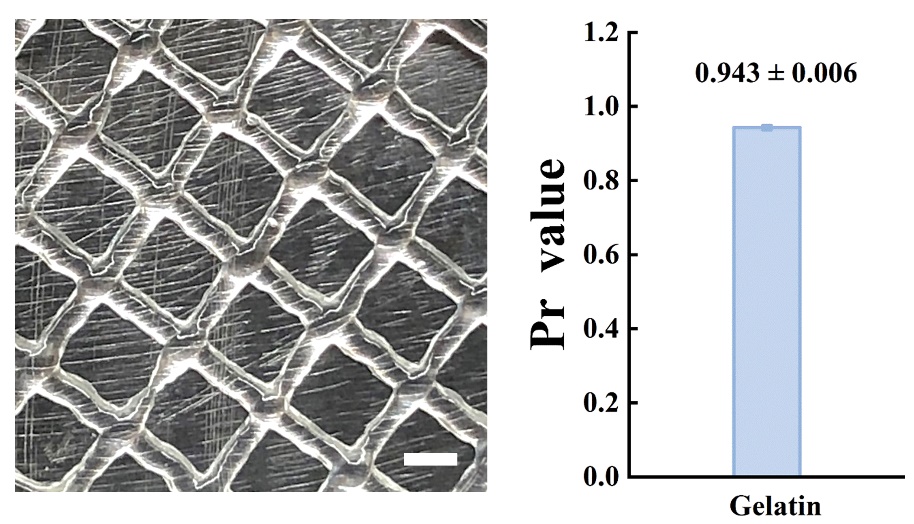


**Fig.S3.** The UV-Vis-NIR spectra of MCBP.


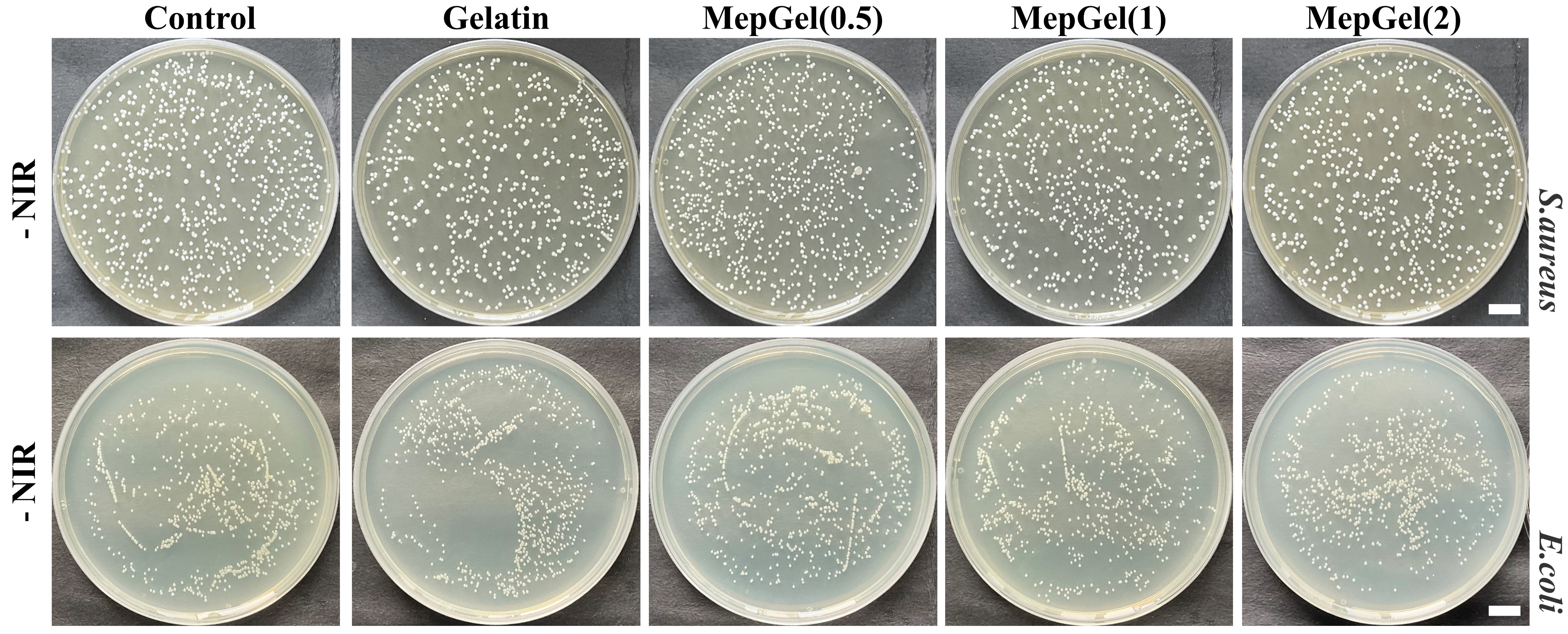


**Fig.S4.** Representative Colony agar plate images after - NIR treatment. Scale bar: 1 cm.

**Fig.S5.** H&E-stained and Masson-stained images of wounds on day 8. Scale bar: 500 μm.


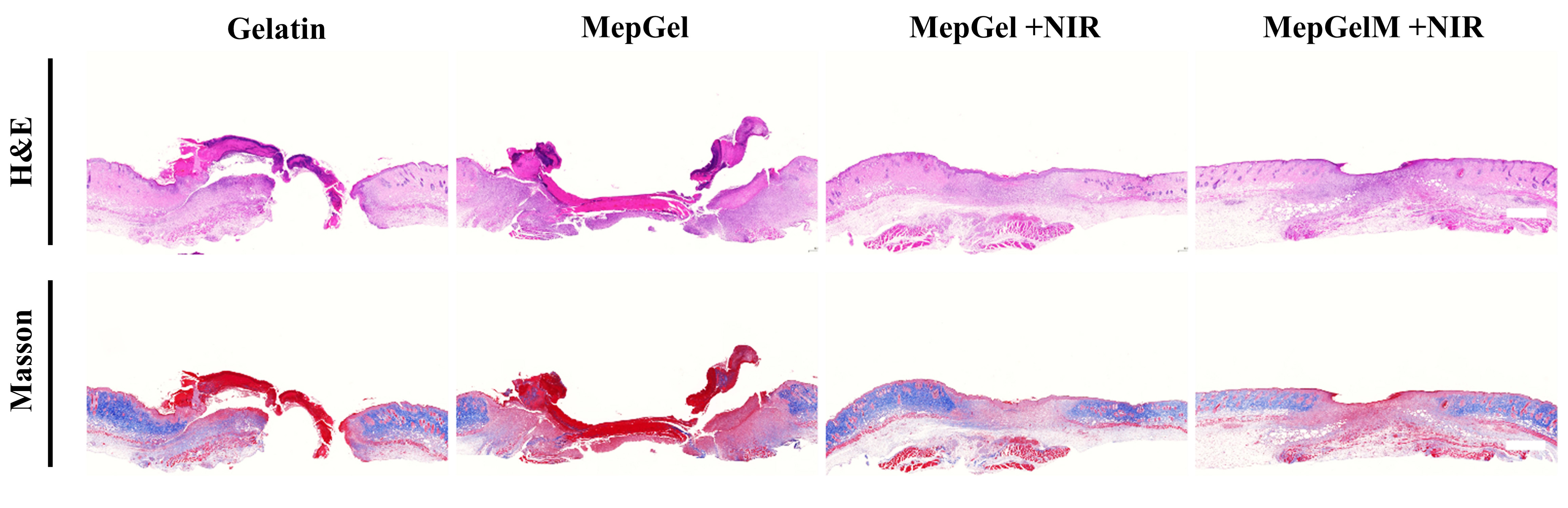


**Table 1.** Basic components, molecular weight, and monosaccharide composition of Mesona chinensis polysaccharide (MCP)


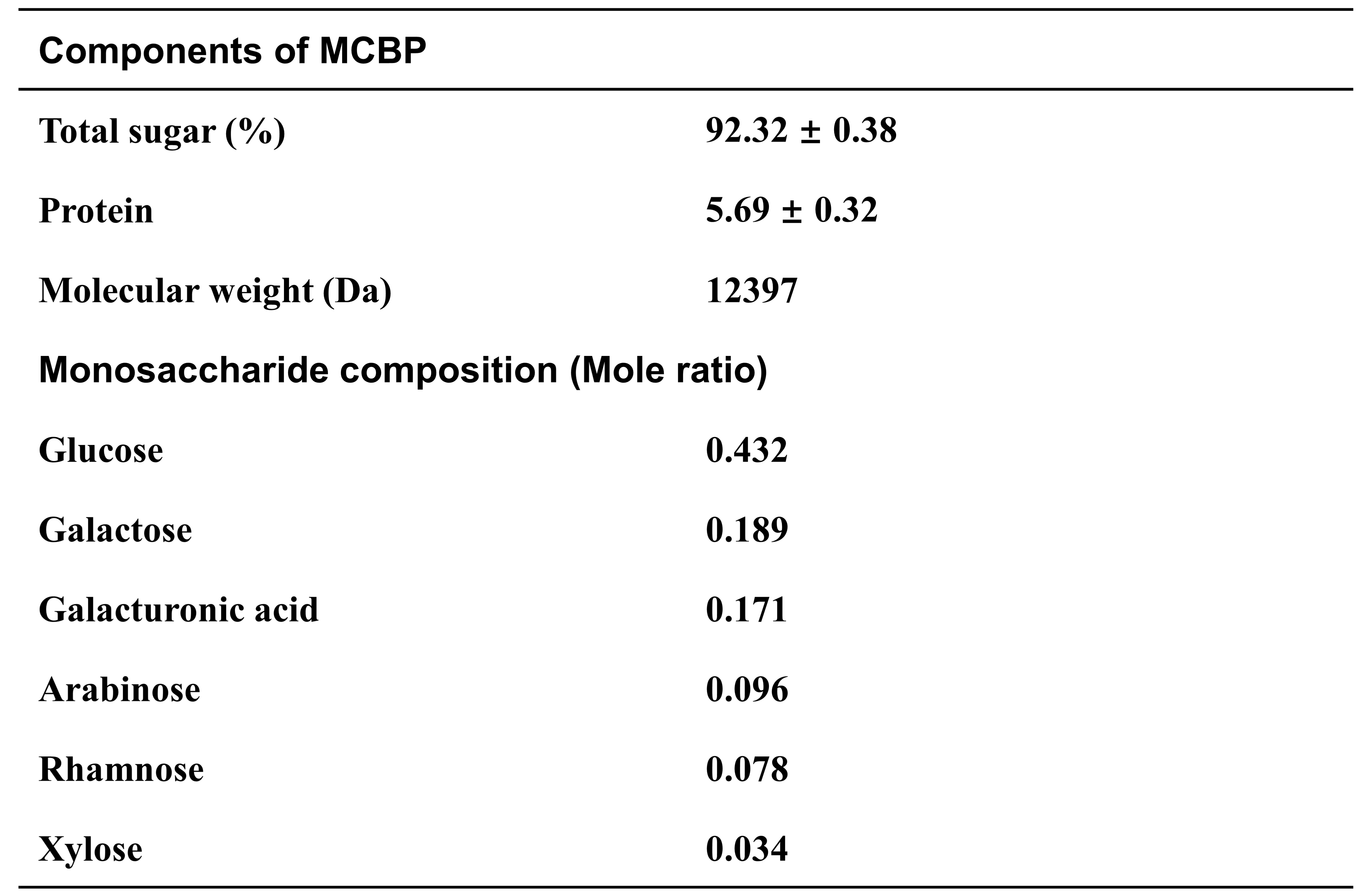


# Video

**Video.S1** The self -healing performance of MepGel(1) hydrogel.

**Video.S2** Near-infrared radiation promoted MepGel(1) hydrogel to cover the wound
